# Supplementary material for: Improved vectors for retron-mediated CRISPR-Cas9 genome editing in Saccharomyces cerevisiae
Source: bioRxiv. 2024 Aug 7:2024.08.06.606807. Preprint. [Version 1] doi: 10.1101/2024.08.06.606807 (PMC11326209; doi:10.1101/2024.08.06.606807)
Supplement: Supplement 2 [file NIHPP2024.08.06.606807v1-supplement-2.pdf]

bioRxiv preprint doi: https://doi.org/10.1101/2024.08.06.606807; this version posted August 7, 2024. The copyright holder for this preprint (which was not certified by peer review) is the author/funder, who has granted bioRxiv a license to display the preprint in perpetuity. It is made available under aCC-BY 4.0 International license.

Supplemental Figures

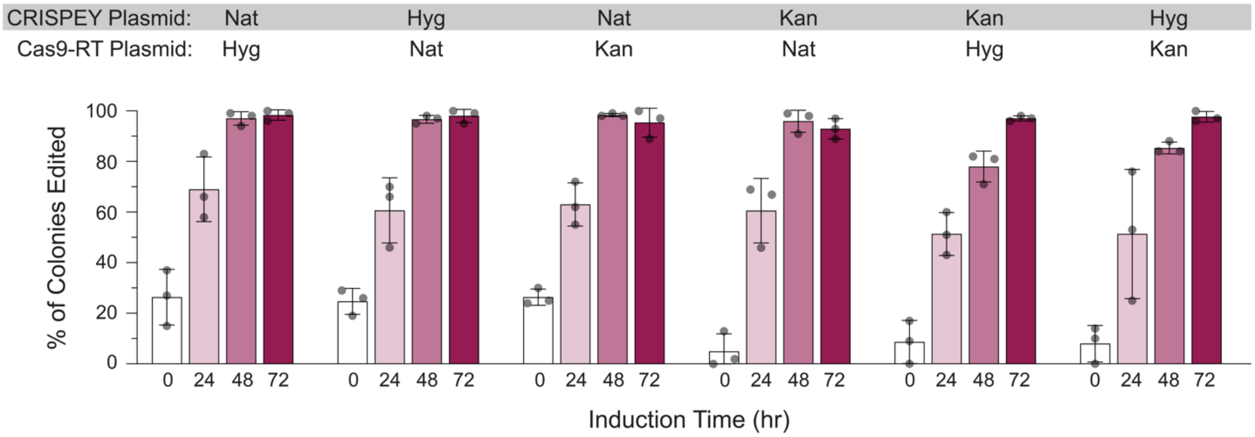

Supplemental Figure 1. The choice selectable marker has a small effect on editing efficiency with the estradiol-inducible CRISPEY system. Cells were transformed with β-estradiol-inducible pCas9-EcRT-Z3 and pCRISPEY-Z3-ADE2 plasmids containing the denoted antibiotic resistance markers. Cells were passaged for the indicated amount of time on β-estradiol-containing selective media and then plated onto YPD to score editing efficiency. Error bars denote the mean and standard deviation of biological triplicates.

bioRxiv preprint doi: https://doi.org/10.1101/2024.08.06.606807; this version posted August 7, 2024. The copyright holder for this preprint (which was not certified by peer review) is the author/funder, who has granted bioRxiv a license to display the preprint in perpetuity. It is made available under aCC-BY 4.0 International license.

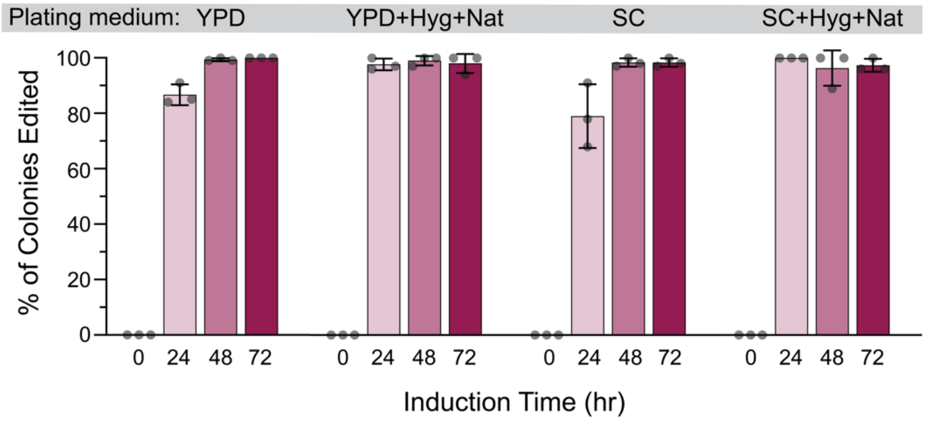

Supplemental Figure 2. Plating medium has little effect on editing efficiency. Cells were transformed with galactose-inducible pCas9-EcRT-GAL and pCRISPEY-GAL-ADE2 plasmids containing the denoted antibiotic resistance markers. Cells were passaged for the indicated amount of time on galactose-containing selective media and then plated onto either non-selective (YPD or SC) media or selective media (YPD or SC plus antibiotics) as indicated to score editing efficiency. Error bars denote the mean and standard deviation of biological triplicates.

bioRxiv preprint doi: https://doi.org/10.1101/2024.08.06.606807; this version posted August 7, 2024. The copyright holder for this preprint (which was not certified by peer review) is the author/funder, who has granted bioRxiv a license to display the preprint in perpetuity. It is made available under aCC-BY 4.0 International license.

Supplemental Tables

Table S1: Reagent, media and chemicals used in this study.

Item	Source	Catalog #
Growth media and components		
Agar	Fisher	BP1423-500
Dextrose (glucose)	Fisher	BP350-1
Galactose	Fisher	AC150615000
Monosodium glutamate (L-glutamic acid; MSG)	Fisher	501804313
Peptone (Bacto)	Fisher	DF0118-17-0
Raffinose	Fisher	AAA1831318
Sodium chloride	Fisher	BP358-10
Tryptone	Fisher	BP1421-500
Yeast extract (Bacto)	Fisher	DF0127-17-9
Yeast nitrogen base w/o amino acids or ammonium sulfate	VWR	90002-646
Enzymes		
BamHI FastDigest restriction enzyme	Fisher	FERFD0054
BglII FastDigest restriction enzyme	Fisher	FERFD0083
DpnI FastDigest restriction enzyme	Fisher	FERFD1704
Eco91I FastDigest restriction enzyme	Fisher	FERFD0394
EcoRI FastDigest restriction enzyme	Fisher	FERFD0274
Mph1103I FastDigest restriction enzyme	Fisher	FERFD0734
MssI FastDigest restriction enzyme	Fisher	FERFD1344
NotI FastDigest restriction enzyme	Fisher	FERFD0595
OliI FastDigest restriction enzyme	Fisher	FERFD1634
PfoI FastDigest restriction enzyme	Fisher	FERFD1754
PvuII FastDigest restriction enzyme	Fisher	FERFD0634
SacI FastDigest restriction enzyme	Fisher	FERFD1134
XhoI FastDigest restriction enzyme	Fisher	FERFD0694
CloneAmp™ HiFi PCR Premix	Takara	639298
Herculase II Fusion DNA Polymerase	Agilent	600675
KOD Hotstart DNA polymerase	VWR	80511-384
Taq Buffer (5x GoTaq)	Fisher	PRM7911
T4 DNA Ligase with 50% PEG4000	Fisher	EL0014
T4 DNA polymerase with NEB Buffer 2.1	NEB	M0203S
Kits		
DNA clean & concentrator-5 kit	Zymo Research	D4013
In-Fusion® HD cloning kit	Takara	638909
NEBuilder® HiFi DNA Assembly cloning kit	New England Biolabs	E5520S
Wizard® Plus Miniprep DNA purification kit	Promega	A1460
Zymoclean DNA recovery kit	Zymo Research	D4007
Chemicals		
Ampicillin	Sigma-Aldrich	A9518
dNTP mix	VWR	95057-676
G418	Gold Biotechnology	G-418-10
Hygromycin B	Gold Biotechnology	H-270-5
Lithium acetate	Fisher	AAA1792130
Nourseothricin Sulfate (ClonNat)	Gold Biotechnology	N-500-1
Polyethylene glycol 3350	VWR	JTU221-8
β-estradiol	VWR	76204-050
Nuclease-free water	IDT	11-05-01-14
Salmon sperm DNA (used to make carrier DNA)	Fisher	NC0448132

bioRxiv preprint doi: https://doi.org/10.1101/2024.08.06.606807; this version posted August 7, 2024. The copyright holder for this preprint (which was not certified by peer review) is the author/funder, who has granted bioRxiv a license to display the preprint in perpetuity. It is made available under aCC-BY 4.0 International license.

Table S2: Primers used in this study.

Primer Name	Sequence (5' - 3')
Amplifies MAT alleles to determine yeast mating type (Illuxley et al, 1990)	
MAT_a_forward	ACT CCA CTT CAA GTA AGA GTT TG
MAT_alpha_forward	GCA CGG AAT ATG GGA CTA CTT CG
MAT_reverse_downstream	AGT CAC ATC AAG ATC GTT TAT GG
Amplifies Ec86-RT and SpCas9 for insertion into PfoI-Mph1103I-digested pAG26 by In-Fusion cloning to create the pCas9-EcRT-GAL-Hyg plasmid (this study)	
Cas9-RT-pAG26_PfoI_F	ACA TGC AGC TCC CGG ATG AAA TTA AAG CCT TCG AGC GTC
Cas9-RT-pAG26_Mph1103I_R	TGA GTT TAG TAT ACA TGC ATG CGA ATT GCA TAT CTT TCC ATA G
Amplifies any MX cassette for swapping markers by homologous recombination (Goldstein & McCusker, 1999)	
PR78 (TEF-F)	CCT TGA CAG TCT TGA CGT GC
PR78 (TEF-R)	CGC ACT TAA CTT CGC ATC TG
Amplifies Z3 promoter for insertion into pCas9-EcRT-GAL plasmids, to replace GAL promoter (this study)	
Z3pr_CAS_Ins_F	TTT ATA TTG AAT TTA TAT TGA ATT TTC AAA AAT TCT TAC TTT TTT TTT GGA TGG ACG
Z3pr_CAS_Ins_R	GGA TCC ACT AGT TTA TAG TTT TTT CTC CTT GAC GTT AAA GTA TAG AGG TAT ATT AAC AA
Amplifies pCas9-EcRT-GAL vectors lacking GAL promoter for Gibson cloning to replace GAL with Z3 promoter (this study)	
Z3pr_CAS_VF	GAA AAA ACT ATA AAC TAG TGG ATC CCC CGG GAA AAA AAT GG
Z3pr_CAS_VR	AAA TTC AAT ATA AAT TCA ATA TAA ATG CCA CCA AAG AAG AAA AGA AAG
Amplifies the Z3EV transcription factor with its ACT1 promoter and 3'UTR for insertion into Mph1103I sites of pCas9-EcRT-Z3pr intermediate plasmids to create final pCas9-EcRT-Z3 plasmids (this study)	
Z3TF_CAS_Ins_F	TTG TGA GTT TAG TAT ACA TGC ATG CGT CGA TCT CCC CTC AAG
Z3TF_CAS_Ins_R	AGA TAT GCA ATT CGC ATG CAT CCC TTT AAA AAC ATA TGC CTC ACC C
Oligo used for Gibson cloning into pCRISPEY-GAL plasmids to swap unique cloning site to XhoI (this study)	
XhoI_swap_CRISPEY	GAG TTA CTG TCT GTT TTC CTC TCG AGG TTT CAG ACG TAT GCT GGA A
Amplifies the XhoI-swapped intermediates of pCRISPEY-GAL plasmids without their GAL promoters for Gibson cloning to insert Z3 promoter and Z3EV transcription factor, creating pCRISPEY-Z3 vectors (this study)	
Z3_CRISP_VF	AAA AAC TAT AAG GGT GCG CAT CTG ATG AG
Z3_CRISP-VR	CGA CGC GAG CGA GCA AAA GGC CAG CAA AAG G
Amplifies the Z3 promoter and Z3EV transcription factor for insertion into the XhoI-swapped intermediates of pCRISPEY-GAL plasmids by Gibson cloning, creating pCRISPEY-Z3 vectors (this study)	
Z3_CRISP_Ins_F	CCT TTT GCT CGC TCG CGT CGA TCT CCC
Z3_CRISP_Ins_R	TGC GCA CCC TTA TAG TTT TTT CTC CTT GAC GTT AAA GTA TAG AGG TAT ATT AAC AA

bioRxiv preprint doi: https://doi.org/10.1101/2024.08.06.606807; this version posted August 7, 2024. The copyright holder for this preprint (which was not certified by peer review) is the author/funder, who has granted bioRxiv a license to display the preprint in perpetuity. It is made available under aCC-BY 4.0 International license.

Amplifies gRNA and repair template for Gibson cloning into any pCRISPEY vector (this study)	
CRISPEY_F	CGG CAT CCT GCA TTG AAT CTG AGT TAC TGT CTG TTT TCC TGG GTC ACG CGT AGG A
CRISPEY_R	ATT TCA ACT TGC TAT GCT GTT TCC AGC ATA GCT CTG AAA C
Sequences gRNA and repair template of any pCRISPEY vector (this study)	
CRISPEY Retron SEQ F	GCA TCT GAT GAG TCC GTG AG
Oligo templates for amplifying specific gRNAs and repair templates to be cloned into any pCRISPEY vector (this study). Repair templates are italicized and gRNAs are bolded.	
ADE2_pos450_CRISPR	GGGTCACGCGTAGGA*GGGTTTTCCATTCGTCTTGAAGTCGAGGACTTTGGCATACGATGGAAGAGCGTAACTTCGTTGTAAAGAATAAGGAAATGATTCCGGAAGCTTTGGAAGTA*AGGAAACCCGTTTCTTCTGACGTAAGGGTGCGCA**ACTTTGGCATACGATGGAAG**GTTTCAGAGCTATGCTGGAA
ADE2_pos444_CRISPR	GGGTCACGCGTAGGA*AGATTTGGGTTTTCCATTCGTCTTGAAGTCGAGGACTTTGGCATACGATGCGAAGAGGTAACTTCGTTGTAAAGAATAAGGAAATGATTCCGGAAGCTTTG*AGGAAACCCGTTTCTTCTGACGTAAGGGTGCGCA**TCGAGGACTTTGGCATACGA**GTTTCAGAGCTATGCTGGAA
CAN1_pos786_CRISPR	GGGTCACGCGTAGGAG*GTTACCGGCCCAGTTGGATTCCGTTATTGGAGAAACCCAGGTGCCTGGGCGTCCAGGTATAATATCTAAGGATAAAAACGAAGGGAGGTTCTTAGGTTGG*AGGAAACCCGTTTCTTCTGACGTAAGGGTGCGCA**TGGAGAAACCCAGGTGCCTG**GTTTCAGAGCTATGCTGGAA
FLO8_164_CRISPR	GGGTCACGCGTAGGA*GAATAGTGAACAGCAGCGACAACAACAGCAGCAGCAGCAACAGCAGCAACATAGAGACAAAGGCCAAAACCCAGTCGACGGACCCAAATCTAAAGAAAAC*AGGAAACCCGTTTCTTCTGACGTAAGGGTGCGCA**GTGGATAAATCAACCTACGG**GTTTCAGAGCTATGCTGGAA
His3delta1_315_CRISPR	GGGTCACGCGTAGGA*CATAGACGACCATCACACCACTGAAGACTGCGGGATTGCTCTCGGTCAAGCTTTGCAGAGGCTAGCAGAATTACCCTCCACGTTGATTGTCTGCGAGGCA*AGGAAACCCGTTTCTTCTGACGTAAGGGTGCGCA**GAGGCCCTAGGGGCCGTGCG**GTTTCAGAGCTATGCTGGAA
HO_CRISPEY_Del	GGGTCACGCGTAGGA*ATGCTTTCTGAAAACACGACTATTCTGATGGCTAACGGTGAAATTAAAGAGCGGGCCTCATAAGAGTTGTGGTAACAACGCAGGTGCGCGCATCTGCTAA*AGGAAACCCGTTTCTTCTGACGTAAGGGTGCGCA**GACGACCAGGTCAGCTAGGG**GTTTCAGAGCTATGCTGGAA
LEU2_CRISPEY_KO	GGGTCACGCGTAGGA*TTTACATTTCAGCAATATATATATATATATTTCAAGGATATACCATTCTAAAAGATTCTCTTTTTTTATGATATTTGTACATAAACTTTATAAATGAAAT*AGGAAACCCGTTTCTTCTGACGTAAGGGTGCGCA**ATTATGGAGAAAAACTGTGG**GTTTCAGAGCTATGCTGGAA
TPS1_KO-#3	GGGTCACGCGTAGGA*CAGGCTAACAAACTAGGTACTCACATACAGACTTATTAAGACATAGAACTTGAACCCGATGCAAATGAGACGATCGTCTATTCCTGGTCCGGTTTTCTCT*AGGAAACCCGTTTCTTCTGACGTAAGGGTGCGCA**TTTCCGGAACCAACATCAAA**GTTTCAGAGCTATGCTGGAA

bioRxiv preprint doi: https://doi.org/10.1101/2024.08.06.606807; this version posted August 7, 2024. The copyright holder for this preprint (which was not certified by peer review) is the author/funder, who has granted bioRxiv a license to display the preprint in perpetuity. It is made available under aCC-BY 4.0 International license.

Table S3: PCR conditions for all amplifications from this study.

Component	Concentration	# of cycles	Condition
Amplification of MAT alleles to determine yeast mating type (Illuxley et al, 1990)			
Taq Buffer (5x)	4 µl	1	95°C for 2 min
dNTPs	200 µM each	30	95°C for 30 sec<br>55°C for 45 sec<br>72°C for 1 min
Mat_a OR Mat_alpha F primer	0.5 µM		
MAT_reverse_downstream primer	0.5 µM		
Genomic DNA template	1 µl	1	72°C for 5 min
Taq polymerase	0.4 µl		
Nuclease-free water	to 20 µl		
Amplification of Ec86-RT and SpCas9 for insertion into PfoI-Mph1103I-digested pAG26 to create the pCas9-EcRT-GAL-Hyg plasmid (this study)			
Herculase II reaction buffer (5x)	5 µl	1	95°C for 2 min
dNTPs	250 µM each	30	95°C for 20 sec<br>53°C for 20 sec<br>72°C for 3 min 10 sec
Cas9-RT-pAG26_PfoI_F primer	0.25 µM		
Cas9-RT-pAG26_Mph1103I_R primer	0.25 µM		
pZS157 DNA	30 ng	1	72°C for 3 min
Herculase II polymerase	0.25 µl		
Nuclease-free water	to 25 µl		
Amplification of MX cassettes (KanMX, HygMX, NatMX) for swapping antibiotic markers by homologous recombination (Goldstein & McCusker, 1999)			
KOD reaction buffer (10x)	5 µl	1	95°C for 2 min
dNTPs	200 µM each	40	95°C for 20 sec<br>50°C for 10 sec<br>70°C for 1 min
$MgSO_4$	1.5 mM		
PR78 (TEF-F) primer	0.3 µM		
PR79 (TEF-R) primer	0.3 µM	1	72°C for 5 min
Genomic DNA template	1 µl		
KOD Hot Start DNA polymerase	1 µl		
Nuclease-free water	to 50 µl		
Amplification of Z3 promoter for Gibson insertion into pCas9-EcRT-GAL plasmids to replace GAL promoter (this study)			
CloneAmp^{TM} HiFi premix (2x)	10 µl	1	98°C for 1 min
Z3pr_CAS_Ins_F primer	0.3 µM	35	98°C for 10 sec<br>55°C for 15 sec<br>72°C for 1 min
Z3pr_CAS_Ins_R primer	0.3 µM		
pRS416-yZ3EV-Z3pr DNA	60 ng		
Nuclease-free water	to 20 µl	1	72°C for 5 min
Amplification of pCas9-EcRT-GAL vectors lacking GAL promoter for Gibson cloning to replace GAL with Z3 promoter (this study)			
Herculase II reaction buffer (5x)	4 µl	1	95°C for 2 min
dNTPs	250 µM each	10	95°C for 15 sec<br>55°C for 20 sec<br>68°C for 5 min 30 sec
Z3pr_CAS_VF primer	0.25 µM		
Z3pr_CAS_VR primer	0.25 µM		
pCas9-EcRT-GAL plasmid DNA	60 ng	20	95°C for 15 sec<br>55°C for 20 sec<br>68°C for 5 min 30 sec<br>(increasing 20sec/cycle)
Herculase II polymerase	1 µl		
Nuclease-free water	to 20 µl		

bioRxiv preprint doi: https://doi.org/10.1101/2024.08.06.606807; this version posted August 7, 2024. The copyright holder for this preprint (which was not certified by peer review) is the author/funder, who has granted bioRxiv a license to display the preprint in perpetuity. It is made available under aCC-BY 4.0 International license.

		1	72°C for 8 min
Amplification of the Z3EV transcription factor with its ACT1 promoter and 3'UTR for insertion into Mph1103I sites of pCas9-EcRT-Z3pr intermediate plasmids to create final pCas9-EcRT-Z3 plasmids (this study)			
CloneAmp™ HiFi premix (2x)	20 μl	1	98°C for 1 min
Z3TF_CAS_Ins_F primer	0.3 μM	35	98°C for 10 sec<br>55°C for 15 sec<br>72°C for 2 min
Z3TF_CAS_Ins_R primer	0.3 μM		
pRS416-yZ3EV-Z3pr DNA	100 ng		
Nuclease-free water	to 40 μl	1	72°C for 5 min
Amplification of the XhoI-swapped intermediates of pCRISPEY-GAL plasmids without their GAL promoters for Gibson cloning to insert Z3 promoter and Z3EV transcription factor, creating pCRISPEY-Z3 vectors (this study)			
Herculase II reaction buffer (5x)	4 μl	1	95°C for 2 min
dNTPs	250 μM each	10	95°C for 15 sec<br>65°C for 20 sec<br>68°C for 5 min 30 sec
Z3_CRISP_VF primer	0.5 μM		
Z3_CRISP_VR primer	0.5 μM		
pCRISPEY-GAL(XhoI) DNA	50 ng	20	95°C for 15 sec<br>65°C for 20 sec<br>68°C for 5 min 30 sec<br>(increasing 20sec/cycle)
Herculase II polymerase	1 μl		
Nuclease-free water	to 20 μl		
		1	72°C for 8 min
Amplification of the Z3 promoter and Z3EV transcription factor for insertion into the XhoI-swapped intermediates of pCRISPEY-GAL plasmids by Gibson cloning, creating pCRISPEY-Z3 vectors (this study)			
CloneAmp™ HiFi premix (2x)	20 μl	1	98°C for 1 min
Z3_CRISP_Ins_F primer	0.25 μM	35	98°C for 10 sec<br>60°C for 15 sec<br>72°C for 1 min
Z3_CRISP_Ins_R primer	0.25 μM		
pRS416-yZ3EV-Z3pr DNA	100 ng		
Nuclease-free water	to 40 μl	1	72°C for 5 min
Amplifies gRNA and repair template for Gibson cloning into any pCRISPEY vector (this study)			
CloneAmp™ HiFi premix (2x)	20 μl	1	98°C for 1 min
CRISPEY_F primer	0.3 μM	35	98°C for 10 sec<br>55°C for 15 sec<br>72°C for 1 min
CRISPEY_R primer	0.3 μM		
gRNA/repair template oligo	100 ng		
Nuclease-free water	to 40 μl	1	72°C for 5 min
